# Questioning the Effectiveness of Oral Cholera Vaccine in Port-au-Prince Slums

**DOI:** 10.4269/ajtmh.16-0314a

**Published:** 2016-08-03

**Authors:** Stanislas Rebaudet, Jean Gaudart, Aaron Aruna Abedi, Renaud Piarroux

**Affiliations:** ^1^ Assistance Publique—Hôpitaux de Marseille, Marseille, France and UMR MD3, Aix-Marseille University, Marseille, France. E-mail: stanreb@gmail.com; ^2^ Assistance Publique—Hôpitaux de Marseille, Marseille, France and UMR 912 SESSTIM (AMU, INSERM, IRD), Aix-Marseille University, Marseille, France. E-mail: jean.gaudart@univ-amu.fr; ^3^ Ministry of Health, Kinshasa, Democratic Republic of the Congo. E-mail: arunaaaron@yahoo.fr; ^4^ Assistance Publique—Hôpitaux de Marseille, Marseille, France and UMR MD3, Aix-Marseille University, Marseille, France. E-mail: renaud.piarroux@ap-hm.fr

Dear Sir:

Oral cholera vaccination (OCV) has been validated by the World Health Organization (WHO) as a valuable tool to complement water, sanitation, and hygiene (WASH) activities in cholera prevention for high-risk areas and populations.^1^ We read with great interest the recent study published by Sévère and others,^2^ which evaluated the effectiveness of a mass OCV campaign targeting approximately 70,000 inhabitants in several slums of Port-au-Prince, Haiti, between April and June 2012. The authors reported a 75% vaccine coverage and, using a cohort design, a striking 97.5% vaccine effectiveness in the 37 months postvaccination, whereas controlled clinical trials have measured OCV vaccine efficacy around 57% [95% confidence interval, 44–67%] during the first 2 years.^3^ Although it was expected that 56% of cholera cases would occur among vaccinated individuals according to the WHO screening method,^4^ the same proportion was 5% in the Sévère and others cohort.

A thorough analysis of this study shows that the authors did not evaluate the isolated effectiveness of OCV. They rather estimated its combined effectiveness together with WASH-associated measures. To assess the importance of such methodological bias, we computed provided data using a bias-indicator cohort analysis, as previously described in another OCV campaign,^5^ and found that their strategy exhibited a 95% effectiveness [93–97%] against noncholeric diarrheas as well. Pondering such bias would require adjusting the results on the observance of WASH prevention methods, which may have differed between nonvaccinated and vaccinated groups.

A cohort study requires that the population be carefully defined and monitored. Conversely, cholera surveillance of both groups was only passively conducted from the GHESKIO (The Haitian Group for the Study of Kaposi's Sarcoma and Opportunistic Infections) cholera treatment center (CTC), and many cholera cases may have been treated elsewhere. During the study period, at least seven CTCs operated in Port-au-Prince within a 5-mile radius around GHESKIO, including three major CTCs operated by Medecins Sans Frontières, and over 20,000 suspected cholera cases were reported to the Haitian Ministry of Public Health and Population.^6^ In addition, the OCV campaign was conducted from April to July 2012, during the main cholera peak of the study period. As the authors started to record cholera cases from April, the cholera attack rate of the nonvaccinated group was overestimated.

Therefore, Sévère and others should have rather conducted a case-control study. Field effectiveness of OCV has previously been evaluated with a test negative case-control design using participant-based analysis with censoring for cholera.^7^ Computing such an analysis using the study data with noncholeric diarrheas as the control group, we found an OCV effectiveness of 67% (41–82%), which is close to the 58% effectiveness (13–80%) of a concomitant OCV campaign conducted in rural Haiti using the same vaccine.^8^

Field reports of OCV campaigns can be interesting to evaluate the feasibility and impact of such strategies. Estimating vaccine effectiveness is also important to detect unexpected programmatic errors. However, vaccine effectiveness results are hampered by many biases that are difficult to ponder in observational studies. Consequently, effectiveness results shall neither be confounded with the experimentally measured vaccine efficacy, nor replace the proper evaluation of vaccine impact on the course of an epidemic.

Finally, as stated by the WHO position paper on cholera vaccines^1^ and suggested by our additional analysis of Sévère and others data, WASH activities remain the corner stone of cholera control and elimination strategies. In Haiti, money is currently lacking to sustain the nationwide reactive program of community awareness and water treatment, and only a tiny fraction of the resources requested by the National Plan for Cholera Elimination in Haiti, 2013–2022^9^ for long-term water and sanitation infrastructures has been pledged so far. Such spectacular but biased OCV effectiveness results shall not even more divert stakeholders and donors from funding these crucial short and long-term WASH programs.
